# Decoding the Effect of Hydrostatic Pressure on TRPV1 Lower-Gate Conformation by Molecular-Dynamics Simulation

**DOI:** 10.3390/ijms23137366

**Published:** 2022-07-01

**Authors:** Muhammad Harith Bin Zamri, Yoshihiro Ujihara, Masanori Nakamura, Mohammad R. K. Mofrad, Shukei Sugita

**Affiliations:** 1 Department of Electrical and Mechanical Engineering, Nagoya Institute of Technology, School of Engineering, Nagoya 466-8555, Japan; harithzamri97@gmail.com (M.H.B.Z.); ujihara.yoshihiro@nitech.ac.jp (Y.U.); nakamura.masanori@nitech.ac.jp (M.N.); 2 Center of Biomedical Physics and Information Technology, Nagoya Institute of Technology, Nagoya 466-8555, Japan; 3 Department of Nanopharmaceutical Sciences, Nagoya Institute of Technology, Graduate School of Engineering, Nagoya 466-8555, Japan; 4 Departments of Bioengineering and Mechanical Engineering, University of California, Berkeley, CA 94720, USA; mofrad@berkeley.edu

**Keywords:** glaucoma, hydrostatic pressure, mechanotransduction, molecular mechanics

## Abstract

In response to hydrostatic pressure, the cation channel transient receptor potential vanilloid 1 (TRPV1) is essential in signaling pathways linked to glaucoma. When activated, TRPV1 undergoes a gating transition from a closed to an open state that allows the influx of Ca^2+^ ions. However, the gating mechanism of TRPV1 in response to hydrostatic pressure at the molecular level is still lacking. To understand the effect of hydrostatic pressure on the activation of TRPV1, we conducted molecular-dynamics (MD) simulations on TRPV1 under different hydrostatic pressure configurations, with and without a cell membrane. The TRPV1 membrane-embedded model is more stable than the TPRV1-only model, indicating the importance of including the cell membrane in MD simulation. Under elevated pressure at 27.6 mmHg, we observed a more dynamic and outward motion of the TRPV1 domains in the lower-gate area than in the simulation under normal pressure at 12.6 mmHg. While a complete closed-to-open-gate transition was not evident in the limited course of our MD simulations, an increase in the channel radius at the lower gate was observed at 27.6 mmHg versus that at 12.6 mmHg. These findings provide novel information regarding the effect of hydrostatic pressure on TRPV1 channels.

## 1. Introduction

Glaucoma is a group of eye diseases that progressively lead to damage to the optic nerve. Although the exact cause of glaucoma has remained elusive, the most common theory suggests that the disease is related to an increase in the fluid pressure inside the eye, referred to as the intraocular pressure (IOP) [1]. The normal range of IOP is between 12–22 mmHg [2] with an average of 14.7 ± 2.8 mmHg [3], and a higher than normal eye pressure is considered to be greater than 22 mmHg [2]. In the human eye, there are over a million retinal ganglion cells (RGCs) in the optic nerve of the retina which processes visual information that it receives from the surrounding photoreceptors in the eye and transmits it to the brain via its associated axons [4]. Previous studies have suggested that elevated IOP levels contribute to the apoptosis of RGCs, initiating the development of glaucoma [5,6]. In-vitro study also supported that tens of mmHg of hydrostatic pressure were enough to cause apoptosis [7] and Ca^2+^ influx [8]. However, how the RGCs sense the IOP and result in apoptosis remains unknown. One theory proposed that “IOP induces physical changes at the Optic Nerve Head (ONH), visualized clinically as optic disc cupping, which causes optic nerve axonal compression at the lamina cribrosa, blockage of axoplasmic flow, and interference in retrograde neurotrophin transport to RGCs, leading to cell death” [6].

**Figure 1:**
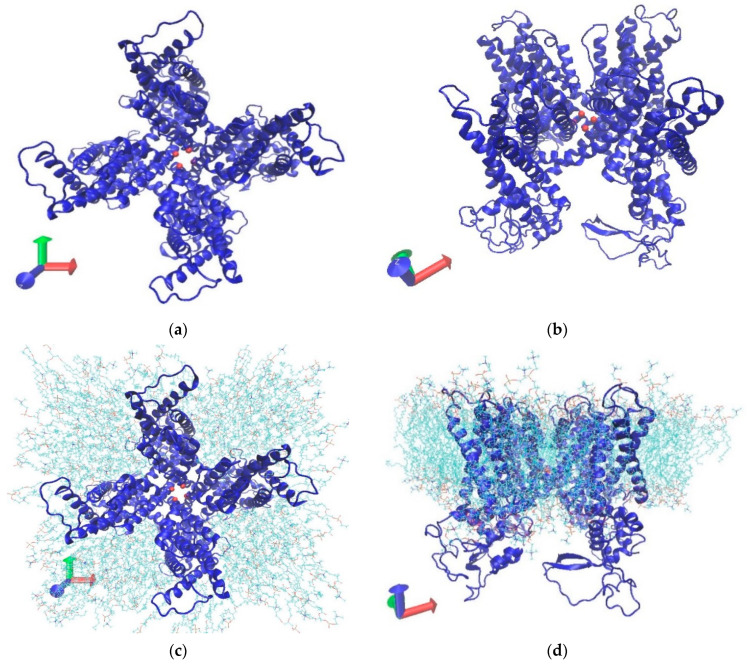
Ribbon diagram of transient receptor potential vanilloid 1 (TRPV1). (**a**,**c**) A top and (**b**,**d**) a side view of (**a**,**b**) TRPV1 only and (**c**,**d**) membrane-embedded models expressed as VMD software. Protein parts are shown as “NewCar-toon” and lipids are shown as “Lines.” For clarity, water and ion molecules have been omitted in this picture. The channel is seen at the center of panels (**a**) and (**c**). Lower gates (I679) are shown as red beads.

One possible mechano-sensor responding to elevated IOP is transient receptor potential vanilloid 1 (TRPV1; see Figure 1). TRPV1 is the first subfamily of the six members of vanilloid receptors under the transient receptor potential (TRP) channels [9]. TRPV1 is vital in signaling pathways associated with various diseases, such as inflammatory diseases [10] and cancer [11], in response to various stimuli such as osmo-chemical stress [12,13]. TRPV1 is highly calcium-permeable [14], and the pore channel is the central site in facilitating ion permeation [12,15,16]. Furthermore, previous studies on the relationship between high concentrations of Ca^2+^ ions and cell death [17,18] have shed light on the linkage between TRPV1 and hydrostatic pressure. In a study conducted by Sappington et al. [8], cell density decreased under elevated hydrostatic pressure, whereas TRPV1 antagonism reduced increases in RGCs intracellular Ca^2+^ concentrations and decreases in cell density. This result indicates that TRPV1 is a mechano-sensor responding to the hydrostatic pressure in RGCs.

TRPV1 exhibits a homo-tetrameric structure that comprises six transmembrane segments (S1–S6) connected by intracellular and extracellular loops, where the pore or ion-conduction pathway forms the re-entrant loop between S5 and S6. In previous studies, the conformational change in TRPV1 structure was apparent in exposure to high temperatures [19,20]. On the other hand, current knowledge on the effect of hydrostatic pressure on TRPV1 is still limited.

This research aims to investigate the relationship between hydrostatic pressure and the TRPV1 conformational state using molecular-dynamics (MD) simulation. To uncover the mechanism of pressure-induced TRPV1 response, the conformational shift at the TRPV1’s pore channel under normal and elevated pressures is observed and compared.

## 2. Materials and Methods

### 2.1. MD Simulation Setup

Molecular dynamics simulations were performed with the GROMACS software [21] version 2020-3 using CHARMM27 force field [22], and a TIP3P water model was used. This research was conducted using two models, the TRPV1-only model and the membrane-embedded model, to examine whether the membrane lipids play a role in affecting the stability or the movement of the TRPV1 atoms. For TRPV1-only simulation, the PDB file of the TRPV1 closed structure (PDB: 5IRZ) was obtained from the Protein Data Bank database [23]. However, since the provided PDB file has missing atoms in the structure, we had to add the missing atoms along with residues 335–751 into the PDB file by using DeepView [24]. For the membrane-embedded model, the same PDB file was used and oriented using Orientations of Proteins in Membranes (OPM) [25] before the embedding process. For the membrane-embedded model, the Membrane Builder function of the CHARMM-GUI web server [26,27] was used to embed TRPV1 in a bilayer of 1-palmitoyl-2-oleoyl phosphatidylcholine (POPC) lipids surrounded by a box of water and ions. Since the 5IRZ model lacks some residues and side chains, the system had a positive net charge. To ensure the electroneutrality of the system and offset the positive net charge, 20 Cl^−^ ions were added. Since this study focuses on channel radius changes, Ca^2+^ ions were not added, meaning that no Ca^2+^ was included in the system. The entire system contains for the TRPV1-only model and TRPV1 membrane-embedded model 354,603 atoms (26,080 = proteins; others = 328,523) and 165,201 atoms (26,080 = protein; 30,016 = POPC; others = 109,105), respectively. An overview of the TRPV1 structure with and without membrane-embedded model can be seen as described in Figure 1 visualized using VMD, wherein water and ion molecules have been removed for clarity [28].

As for the energy minimization phase, harmonic restraints with a force constant of 1000 kJ/mol/nm^2^ were applied with 50,000 steps of the Steepest descent method. The same harmonic restraints with a force constant of 1000 kJ/mol/nm^2^ were applied for equilibrations and MD runs. After energy minimization was completed, temperature (*T* = 37 °C) and pressure equilibrations were performed for 100 ps (2 fs × 50,000 steps), respectively [29]. We applied 12.6 and 27.6 mmHg as gauge pressure, that is, 1.03 and 1.05 bar, respectively, in the absolute pressure. Finally, production MD runs were generated in the NPT ensemble. The Nose–Hoover method [30] was used with the temperature kept constant at *T* = 37 °C. The Parrinello–Rahman method [31] was used for pressure coupling. The Ewald process particle mesh [32] was used for electrostatic calculations. Both simulations were conducted for 100 ns using 2 fs time steps. The computation was carried out using the supercomputer "Flow" at Information Technology Center, Nagoya University. Our molecular-dynamics simulations were run on a FUJITSU Server PRIMERGY CX2570 M5 (Fujitsu, Tokyo, Japan) with the Intel Xeon Gold 6230 CPU (20 core, 2.10–3.90 GHz × 2 sockets) and it took 3 days for the 100 ns simulation time.

### 2.2. Root Mean Square Deviation (RMSD) Analysis

In order to determine the conformational stability of TRPV1, the root mean square deviation (RMSD) of the Cα atoms, the first carbon atom that attaches to a functional group in each amino acid, was determined relative to the initial structure for each of the four sets (TRPV1-only model at normal pressure and elevated pressure levels, TRPV1 membrane-embedded model at normal and elevated pressure levels). In total, there were 10,000 timeframes of data snapshots obtained from the trajectory output file for each set. The RMSD of each set was calculated by least-square fitting the structure to the reference structure ($r_{i}^{ref}$), as detailed in Equation (1) [33]:

(1) $$RMSD\left(t\right)={\left[\frac{1}{M}\overset{N}{\underset{i=1}{\sum }}m_{i}{\left|r_{i}\left(t\right)-r_{i}^{ref}\right|}^{2}\right]}^{\frac{1}{2}}$$

where $M=\overset{N}{\underset{i=1}{\sum }}m_{i}$, *N* is the number of atoms, *m*_i_ is the mass of atom *i*, and $r_{i}\left(t\right)$ is the position vector of atom *i* at time *t* after least square fitting the structure to the reference structure. To assess the equilibration of each simulation, the time for RMSD to reach 90% of its asymptotic value was determined using the first-order lag function:

(2) $$RMSD\left(t\right)=a\left(1-e^{-t/\tau }\right),$$

where *a* and *τ* are fitting constants. The actual RMSD values were compared with the fitted values of the first-order lag function. Then, the average RMSD for each simulation was calculated after the RMSD reached 90% of its asymptotic value.

### 2.3. Radius of Gyration Analysis

To probe the domain movement at the lower gate, the radius of gyration (*R_g_*) was calculated as described in Equation (3):

(3) $$R_{g}=\sqrt{\left(\frac{\sum_{i}{\left|x_{i}\right|}^{2}m_{i}}{\sum_{i}m_{i}}\right)},$$

where *m_i_* is the mass of atom *i* and ***x****_i_* is the position of atom *i* with respect to the center of mass of the molecule. *R_g_* analysis offers a simple and intuitive way to probe the symmetric contraction/expansion motions that could effectively assist in describing the stability of proteins.

### 2.4. Radius Analysis

To elucidate the effect of hydrostatic pressure on the conformational shift in TRPV1, the radii at the lower gate of the pore channel for all four sets were calculated using the HOLE program [34]. The average radius for each simulation was then calculated after the RMSD reached 90% of its asymptotic value determined using the first-order lag function.

### 2.5. Statistical Analysis

We evaluated the variations with F-test and the averages with t-test for the RMSD, *R_g_*, and radius at the lower gate between the simulations. The significance level of *p* = 0.05 was used. Data are shown as mean ± standard deviation (SD).

## 3. Results

Data in this study can be obtained from Supplementary Materials (Data1).

### 3.1. RMSD

**Figure 2:**
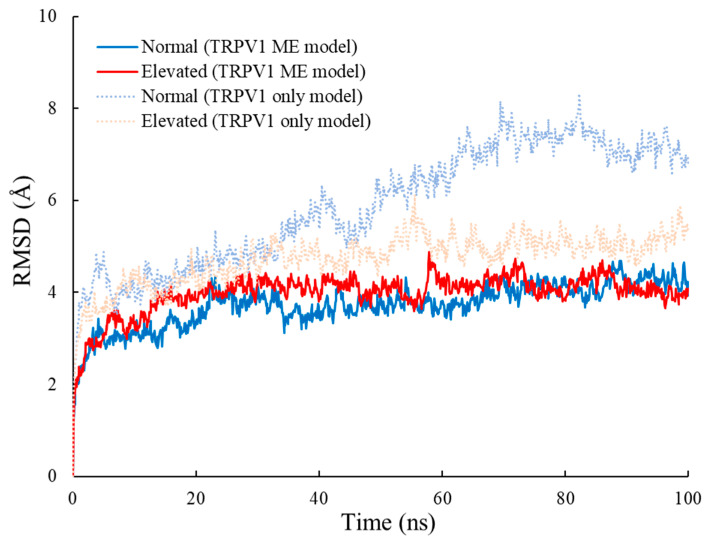
Root mean squared deviation (RMSD) of TRPV1. Molecular dynamics (MD) simulated at normal (12.6 mmHg) and elevated (27.6 mmHg) hydrostatic pressure are shown in blue and red, respectively, using TRPV1-only and TRPV1 membrane-embedded (ME) models. Data are plotted using all 10,000 timeframes obtained from the output trajectory files.

The RMSD of the TRPV1-only model and the TRPV1 membrane-embedded model at normal and elevated pressure levels is shown in Figure 2. The graph patterns for the TRPV1-only model showed that both graphs fluctuated almost similarly (±1 Å) until about 35 ns. The RMSD graph pattern at normal pressure started to deviate more substantially than that at the elevated pressure level, where a large difference between the two graphs can be seen starting from approximately 60 ns until the end of the simulations. In addition, we found that the TRPV1-only model simulation at normal pressure took 39.0 ns to reach 90% of its asymptotic value as compared to 4.9 ns at elevated pressure. As for the graph patterns for the TRPV1 membrane-embedded model, both graph patterns fluctuated almost similarly (±1 Å) until the end of the simulations. The TRPV1 membrane-embedded model under normal and elevated pressure took 6.1 and 6.7 ns, respectively, to reach 90% of its asymptotic value. Therefore, the RMSD results indicate that all simulations reached 90% of their asymptotic values early, and a 100 ns timeframe is sufficient to obtain a stable state of the proteins.

In the TRPV1-only model, the average RMSD at normal pressure (6.8 ± 0.7 Å), calculated for the timeframes starting from 39.1 ns, was slightly higher than that at elevated pressure (5.0 ± 0.3 Å), with a significance level of *p* = 0.05. This result indicates that the TRPV1-only model fluctuated largely at normal pressure than elevated pressure. In the TRPV1 membrane-embedded model, the average RMSD at normal pressure (3.8 ± 0.4 Å), calculated for the timeframes starting from 6.8 ns, was lower than that at elevated pressure (4.1 ± 0.3 Å), with a significance level of *p* = 0.05. Contrary to the TRPV1-only model, the TRPV1 membrane-embedded model fluctuated largely at elevated pressure than normal pressure. Taken together with these results, we concluded that fluctuation magnitude is not determined by the hydrostatic pressure level.

Comparing the RMSD values between models, they exhibited larger deviations in the TRPV1-only model as compared to those observed in the TRPV1 membrane-embedded model for both normal and elevated pressures. This result implies that the membrane suppresses the fluctuation of proteins. Therefore, the membrane that is a more physiologically representative model should be considered for the MD simulations of this membrane protein.

### 3.2. Radius of Gyration

**Figure 3:**
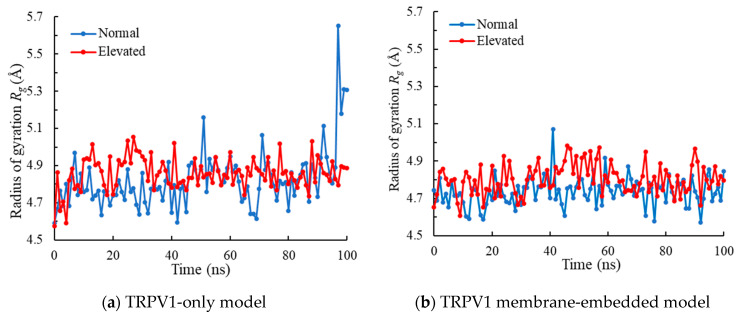
The radius of gyration *R_g_* at the lower gate of (**a**) TRPV1-only model and (**b**) TRPV1 membrane-embedded model.

The radius of gyration *R_g_* values at the lower gate of the TRPV1-only and membrane-embedded models at normal and elevated pressure are shown in Figure 3. In the TRPV1-only model, the average *R_g_* at the lower gate did not exhibit an appreciable difference between the normal vs. elevated pressure (4.86 ± 0.18 Å and 4.86 ± 0.07 Å, respectively). In the TRPV1 membrane-embedded model, the average *R_g_* at the lower gate at normal and elevated pressure was 4.74 ± 0.08 Å and 4.80 ± 0.08 Å, respectively, and *R_g_* at elevated pressure was slightly higher than those at normal pressure (statistical significance *p* < 0.05). This result indicates that the lower gate shows larger outward motions at elevated pressure than normal pressure.

### 3.3. Radius of Lower Gate

**Figure 4:**
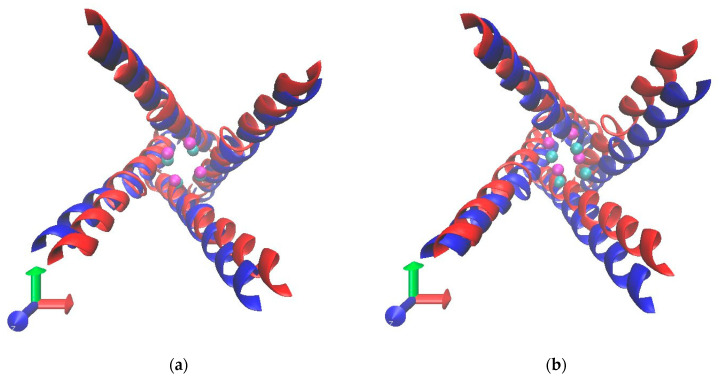
A top view of the gate of TRPV1 membrane-embedded models at normal (blue) and elevated pressure levels (red) at (**a**) 47.5 and (**b**) 100 ns timeframe. Only S6 domains (residues from 659-689) are shown as a ribbon diagram. The lower gate (I679), shown as beads, is colored in cyan and magenta at normal and elevated pressures, respectively.

A comparison of the radius of the lower gate of TRPV1 membrane-embedded models at normal vs. elevated pressure levels are shown in Figure 4a and Video S1. The figure shows a screenshot taken at 47.5 ns depicting the expansion of the lower gate under high pressure focusing on the backbones of the lower gates, though the difference was small. Mostly, the gate radii from the images were too difficult to compare because the lower gate positions of the two models were not the same as shown in Figure 4b, which was captured at the final moments of the simulation (100 ns).

**Figure 5:**
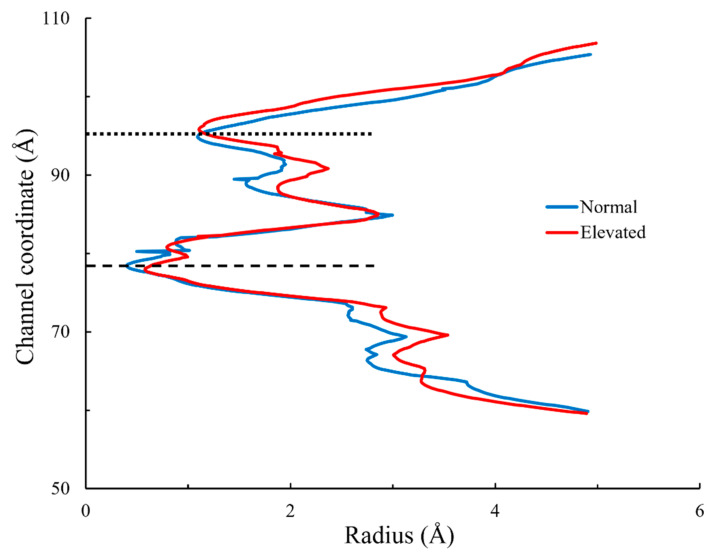
Pore-channel radius at 100 ns timeframe. Pore-channel radii at normal and elevated hydrostatic pressures are shown in blue and red, respectively. The dashed and dotted lines indicate the position of the TRPV1 lower and upper gates, respectively.

The radius of the pore channel (residues 630–687) of the TRPV1 membrane-embedded model is shown in Figure 5 (see Video S2 for whole 100 ns). The radii corresponding to every atom coordinate along the axis of the pore channel at residues 630–687 were extracted from the final timeframe of 100 ns for the two pressure conditions. Comparing the pore-channel radius at normal and elevated pressure, it can be observed that there was an increase in the overall size of the pore channel under elevated pressure.

The distance between lower and upper gates was also analyzed to investigate the conformational changes in the S6 domain. The distance was 16.0 ± 1.1 Å and 17.1 ± 2.1 Å for normal and elevated pressures, respectively. The distance at the elevated pressure is slightly, but statistically significantly (*p* < 0.05), higher than that at the normal pressure. This result indicates that the pore channel is stretched locally along the channel axis under elevated pressure.

**Figure 6:**
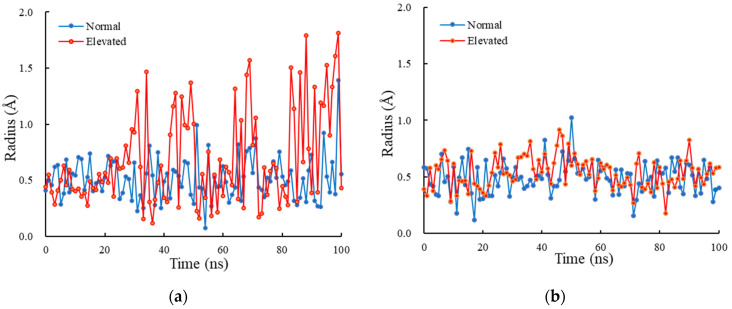
The lower-gate radius at normal and elevated hydrostatic pressure. (**a**) TRPV1-only model and (**b**) TRPV1 membrane-embedded model.

The lower gate radius of the TRPV1-only model and the TRPV1 membrane-embedded model were then compared at normal and elevated pressure (see Figure 6). Based on the equilibration of the simulations, we calculated the average radius starting from 39 ns for the TRPV1-only model and 7 ns for the TRPV1 membrane-embedded model. From the data plotted in Figure 6a, the average lower gate radius of the TRPV1-only model at normal and elevated pressure was 0.52 ± 0.22 Å and 0.78 ± 0.47 Å, respectively. The average radius at elevated pressure was higher than that at normal pressure, with a 50% change in average radius between the two pressure levels. Both F-test and t-test indicated that the difference in variance and average radius between the two pressure levels was statistically significant. The average lower-gate radius of the TRPV1 membrane-embedded model at normal and elevated pressure was 0.49 ± 0.14 Å and 0.54 ± 0.14 Å, respectively (see Figure 6b). The radius at elevated pressure was slightly, but statistically significantly (*p* < 0.05), higher than that at normal pressure, with a 10% change in average radius. Both models show an increase in the lower-gate radius at elevated pressure compared to normal pressure.

## 4. Discussion

The lower gate of the TRPV1 is located at residue ILE 679 (I679) and is the narrowest part of the pore channel (Figure 5). This result is consistent with the findings by Liao [35], which describe the lower gate of the TRPV1 as the most constricted point of the pore channel. The increase in the radius of the lower gate under elevated pressure in both models is evident from our results because the difference is statistically significant, though the increase in the lower-gate radius is not large. This result indicates that the elevated hydrostatic pressure influences the increase in the radius of the lower gate of TRPV1. Moreover, the difference in absolute pressure we used was not very large: normal and elevated pressures were 1.03 bar (12.6 mmHg as gauge pressure) and 1.05 bar (27.6 mmHg as gauge pressure), respectively. Since we set such pressure levels from physiological pressure, the physiological IOP increase is likely to enlarge the lower gate of TRPV1. An increase in hydrostatic pressure, much like an increase in temperature, perturbs the thermodynamic equilibrium between the native and unfolded states of proteins [36,37]. The differences in radius under different pressure states showed how elevated pressure causes movement at the lower gate.

In the TRPV1 membrane-embedded model, the radius of gyration *R_g_* at the elevated pressure was statistically higher than that at the normal pressure, with a significance level of *p* = 0.05. The *R_g_* is an indicator of how compact a protein is [38,39,40] and is a concern with how normal secondary structures can be compactly packed into a 3-dimensional structure of proteins. On top of that, *R_g_* is also used to probe the inward/outward domain motions of a protein at residue level [20]. Therefore, increases in the *R_g_* at the lower gate of the TRPV1 membrane-embedded model at elevated pressure (Figure 3b) indicates that there are larger outward motions at the lower-gate domains than normal pressure. In Figure 3b, we can see the fluctuation in the radius of gyration. The fluctuation shows the large outward/inward movement of the lower gate, which might show the short time opening of this channel. Since the ions might pass through the lower gate even in a short time, a larger channel radius under elevated pressures has the advantage of passing ions through TRPV1.

The distance between lower and upper gates is larger under the more elevated than normal pressure. This result was supported by Cao et al [41], in which the distance was ~15 Å and seemed to increase after the activation of TRPV1 with capsaicin bounding. Thus, our result indicates pressure elevation contributes not only to the opening of the lower gate but to stretching the gate axially with conformational changes in the channel of TRPV1. Reportedly, multiple domains in TRPV1 move under high temperatures [19], which might also contribute to an increase in the distance between the gates and ion flux.

Despite the observation and change in the lower gate radius of TRPV1, we did not observe a complete gating transition from a closed state to opened state (activated state) within the 100 ns simulation time. One of the reasons we did not see the gate transition could be a shortage of simulation time. Conformational change such as TRP channel gaiting takes a microsecond to millisecond of time [42]. When considering the ability of ions to perturb molecular conformation, certain factors such as the structural characteristics of the pores, the hydrophobicity and the afforded diameter of the conduction pathway, must be taken into account. For example, ions can move through in partially or fully dehydrated states with a lower bond ionic radius of approximately 1 Å for fully dehydrated Na^+^ or Ca^2+^ ions [43], which is a comparable value of the lower gate radius (~1 Å at the maximum) in our simulation (Figure 6b). The lower gate of TRPV1 can only be considered open when it is large enough to allow the permeation of extracellular ions. Other factors are required to fully change the gating transition from a closed to opened state. We used PDB:5IRZ in this study, and the model lacked residue 110–334 containing the ARD linker and ankyrin chains, which might play a pivotal role in the binding of external ions [44]. Although the gate radius change is not large enough for ions to pass through, increases in the radius and outward movement of the lower gate under elevated pressure present the symptom of the gate opening.

Some membrane proteins change their structure due to the application of external force. For example, the TREK subfamily protein changes its structure and passes K^+^ ions. It is well-known that lateral forces acting on the channel protein are the most important to causing gate transition [45,46] and are produced by the stretch, bending, shearing, torsion, and local effects of the channel protein [45]. Although hydrostatic pressure is not included in these forces, hydrostatic pressure might also affect the changes in lateral force (or strains).

The RMSD for the membrane-embedded model simulation under the two different pressures were both observed to be lower than those in the TRPV1-only model (Figure 2). A low RMSD value indicates that the protein deviates less from its initial structure throughout the simulation [47]. We believed that the addition of a membrane bilayer plays a role in affecting protein stability during simulations. When the TRPV1 is embedded in a bilayer membrane, the protein should be constrained and held together in place by the surrounding POPC molecules. If that is the case, this causes the movement of TRPV1 atoms in the bilayer membrane to be more limited. In vivo, there is a membrane around the TRPV1. Given that the results that RMSD and *R_g_* are different between models, the membrane should be included in MD simulation.

Of course, the limitations of this study are also apparent. As such, the constituents of the phospholipid used for the membrane-embedded model (POPC) might not be fully accurate. In fact, there are other phospholipid models that have not been tested for MD simulations to this day. Ion permeation from extracellular to intracellular spaces was not directly observed, and Ca^2+^ ion flux observation is necessary to evaluate gate function. To reduce simulation time, setting Ca^2+^ around the lower gate and calculating the time to pass the gate might be useful. Since this research was conducted from the closed state, it is also necessary to repeat this study using an open-state TRPV1 to compare and understand the effect of elevated pressure on the close-to-open-state transition of the lower gate of TRPV1. Finally, this study did not observe a full transition from close to open states. In the limited simulation, the higher pressure condition is a useful method to enhance the transitional change in the TRPV1 lower gate. In fact, in a study investigating the gate transition of TRPV1 due to temperature changes using MD, higher temperatures beyond the physiological state showed heat-activated conformational changes in the outer pore of TRPV1 [19]. These are subject to our future studies.

## 5. Conclusions

The aim of this study was to explore the relationship between hydrostatic pressure and TRPV1 lower-gate conformation using MD simulation. Comparing the results of the TRPV1-only model vs. the TRPV1 membrane-embedded model suggested that a membrane is required for a more physiologically representative simulation. Although we did not observe the full conformational transition from closed to open state within the course of a 100 ns MD simulation, our results suggested that the lower-gate radius at elevated hydrostatic pressure is larger than that at normal pressure, indicating that the hydrostatic pressure influences the state of gating transition. The effect of hydrostatic pressure on TRPV1 is still unclear. More extensive molecular dynamics simulations are needed in the future to provide more insight into the effect of hydrostatic pressure on TRPV1.

## Data Availability

The data presented in this study are available in Supplementary Materials.

## Supplementary Materials

The following supporting information can be downloaded at: https://www.mdpi.com/article/10.3390/ijms23137366/s1.
