# Supplementary figures and images for: Decoding the Effect of Hydrostatic Pressure on TRPV1 Lower-Gate Conformation by Molecular-Dynamics Simulation
Source: Int J Mol Sci. 2022 Jul 1;23(13):7366. doi: 10.3390/ijms23137366 (PMC9266826; doi:10.3390/ijms23137366)

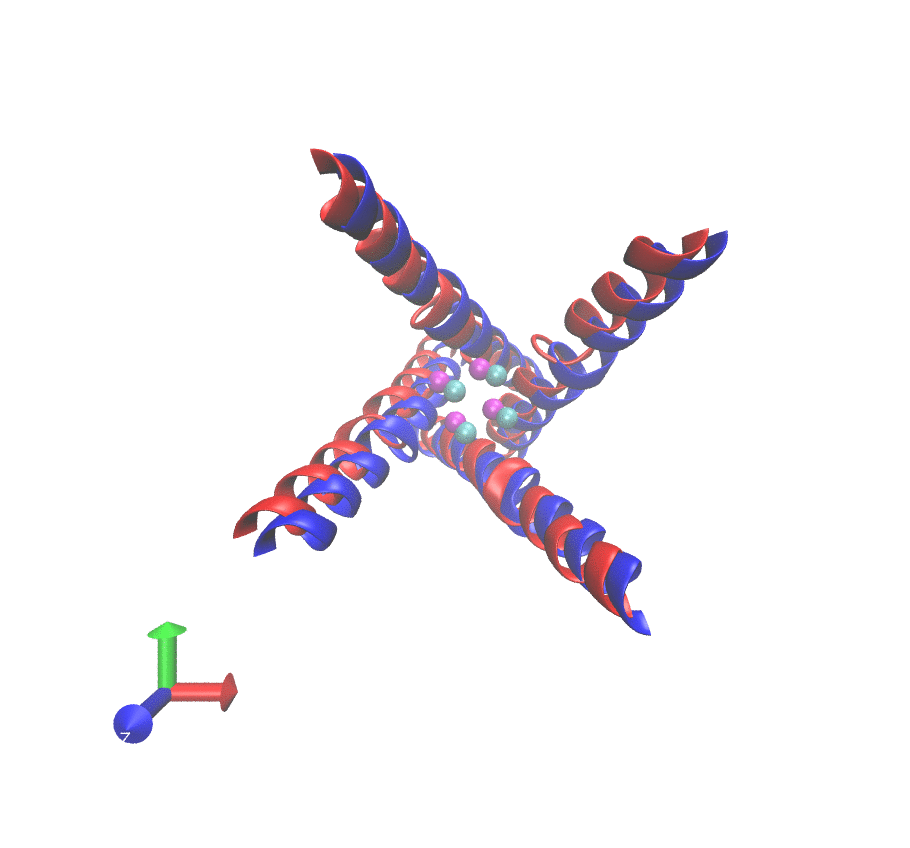

Supplement: Supplementary file 1 [file ijms-23-07366-s001.zip › Movie S1.gif]

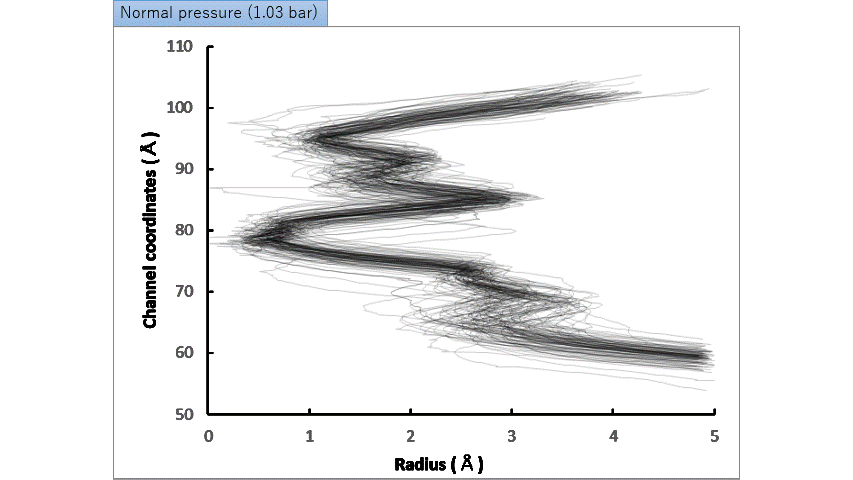

Supplement: Supplementary file 1 [file ijms-23-07366-s001.zip › Movie S2.gif]
